# Supplementary material for: Deterioration of modern concrete structures and asphalt pavements by respiratory action and trace quantities of organic matter
Source: PLoS One. 2021 May 13;16(5):e0249761. doi: 10.1371/journal.pone.0249761 (PMC8118311; doi:10.1371/journal.pone.0249761)
Supplement: S1 File — (DOCX) [file pone.0249761.s001.docx]

**Supporting information**

**S1 Fig 1. Volume expansion and delamination (cracks: white arrow) of new mortar samples**

Fig 1A. Volume expansion of sample A (Nittetsu cement; 6%).

Fig 1B. Volume expansion of sample B (extracted Nittetsu cement; 0% ).

Fig 1C. Volume expansion of sample C (extracted cement including AE water-reducing agent; 9.4%).

Fig 1D. Volume expansion of sample E (Kazakhstan cement including AE water reducing agent; 6.1%).

Fig 1 shows volume expansion of four samples (samples A, B, C, and D) at 30 days after placement with the red lines on the samples showing the original height of the sample. White arrows in Fig 1 show the delamination (cracks) in the samples.

**S2 Fig. 2. Amorphous substances (black) and disaggregation in amorphous substances in new mortar samples**

Fig 2 A. Amorphous substance of sample A (Nittetsu cement)

Fig 2 B. Amorphous substance of sample B (Extracted cement)

Fig 2 C. Amorphous substance of sample C (Extracted cement + AE water reducing agent)

Fig 2 D. Amorphous substance of 120 year-sample

Fig 2 shows that the disaggregation appears as shown out-side the fine particles (tips of white arrows) in the amorphous substances (black).

Figs 2A. - 2D. show the two-dimensional CT images on the surface of samples at the center of samples 30 days after its placement. The white fine particles are independent and the outside of the white fine particles show as black. It shows that disaggregation occurred at the outside of the white fine particles in the samples.

**S3 Fig. 3 Distribution of three dimensional-cracks in new mortar sample A and the 120 year-sample.**

Fig 3A. Crack widths from 0.05 mm (red) to 0.25 mm (purple) in the 120 year-sample.

Fig 3B. Crack widths from 0.138 (red) to 0.552 mm (purple) in mortar sample A.

Fig 3A and Fig 3B show the crack width and crack length of the sample of 120 year-sample and mortar sample A. Fig 3 shows that the length of cracks (1 mm and/or longer) in sample A was longer than in the 120 year-sample.

**S4 Fig 4. Cracks in the damaged aggregate and crack distribution of three-dimensional cracks of the samples in the first (0-2.5 cm) and second layers (3-5.5 cm)**

Fig 4A and Fig 4B The cracks in the aggregate and cracks in the mortar in the first layer.

Fig 4C and Fig 4D The cracks in the aggregate and cracks in the mortar in the second layer.

Fig 4A and Fig 4B show the cracks in the aggregate and cracks in the mortar in the first layer. Fig 4C and Fig 4D show the cracks in the aggregate and cracks in the mortar in the second layer.

**S5 Fig 5. Distribution of organic matter contents of concrete slab, foundations of handrail, and bridge supporting pier**

Fig 5 shows the ratio of the content of organic matter to mortar for the concrete slab, foundation of the handrail, and bridge supporting pier.

No.1 is for the upper part of the foundations of the hand rail, No. 2 is for the bottom part of the foundations of the hand rail. The samples of the bridge pier were collected from the same bridge pier, and the air means the part of the bridge pier (air) exposed to the air, and the water means the part of the bridge pier (water) that was submerged in the river.

**S1 Table 1. The degree of deterioration (DD: %) in mortar samples, content of DBP in cement and /or mortar, and maximum expansion of samples (%)**

Table 1 shows the degree of volume expansion and DD values for mortar samples (2 x 2 x 2 cm) made of the 11 types of cement. Here, the extracted Nittetsu cement shows cement with TQOM extracted from Nittetsu cement using chloroform and Soxhlet extractor.

**S2 Table 2 Properties of 120 year-sample and new mortar sample A (30 days after formation) for seven factors ((1)-(7)) of crack and deterioration**

Table 2 shows the crack properties (crack width), void properties and ratios of crack lengths of specific crack widths to the total crack length in the 120 year-sample and sample A. Table 2 shows the index of the main damage for mortar sample is the wider crack width and crack length of the wider crack width rather than deterioration (DD value) for damage evaluation of mortar samples.

**S3 Table 3. Main chemical components, emission sources of the organic components of samples in disaggregated cement concrete, TSM, DEP, tire debris, and asphalt (bitumen)**

Table 3 shows that TSM contained phthalates, amine compounds, and SPNES. These suggest that it is trace quantities of harmful organic matter in TQOM that cause damage to asphalt pavements and concrete structures (T. Tomoto, Building and Environment, 44(2009) 2000-2005[3]).

**S4 Table 4. Properties of asphalt mixtures in runway in Nagoya**

Table 4 shows that the amount of these residual water contents (water content) after the transient moisture permeation test for core samples (surface course and binder course were added: 1998) of three areas of the asphalt runway (water content: blistering; 6.5%, rutting; 4.7%, runway in service; 15.5%) was larger than the water content (0.6%) in the core sample of the highway.
