# Supplementary material for: Deterioration of modern concrete structures and asphalt pavements by respiratory action and trace quantities of organic matter
Source: PLoS One. 2021 May 13;16(5):e0249761. doi: 10.1371/journal.pone.0249761 (PMC8118311; doi:10.1371/journal.pone.0249761)
Supplement: Attachment — Submitted filename: PLOS ONE_Response to revierwes.pdf [file pone.0249761.s002.pdf]

Response to Reviewers

Additional Requirements:

When submitting your revision, we need you to address these
Additional requirements.

1. Please ensure that your manuscript meets PLOS ONE's style requirements, including those for file naming. The PLOS ONE style templates can be found at

We read again the Submission Guidelines of PLOS ONE.

We revised the name order, Introduction and Conclusions according to PLOS ONE' style.

https://journals.plos.org/plosone/s/file?id=wjVg/PLOSOne_formatting_sample_main_body.pdf

and

https://journals.plos.org/plosone/s/file?id=ba62/PLOSOne_formatting_sample_title_authors_affiliations.pdf

2. Please ensure that in your methods section you have provided details of the sources of all materials, chemicals, equipment and instrumentation used in your study, including manufacturer names where relevant. This is in line with our reproducibility criterion for publishing, see https://journals.plos.org/plosone/s/criteria-for-publication#loc-3

We revised the methods with materials, chemicals, equipment and instrumentation. These sentences shorten Specially, we revised for ^{1}H NMR test method in text.

3.We note that you have indicated that data from this study are

available upon request. PLOS only allows data to be available upon request if there are legal or ethical restrictions on sharing data publicly. For more information on unacceptable data access restrictions, please see http://journals.plos.org/plosone/s/data-availability#loc-unacceptable-data-access-restrictions.

We showed the data in this study as possible as they can.

In your revised cover letter, please address the following prompts:

a) If there are ethical or legal restrictions on sharing a de-identified data set, please explain them in detail (e.g., data contain potentially sensitive information, data are owned by a third-party organization, etc.) and who has imposed them (e.g., an ethics committee). Please also provide contact information for a data access committee, ethics committee, or other institutional body to which data requests may be sent.

b) If there are no restrictions, please upload the minimal anonymized data set necessary to replicate your study findings as either Supporting Information files or to a stable, public repository and provide us with the relevant URLs, DOIs, or accession numbers. For a list of acceptable repositories, please see http://journals.plos.org/plosone/s/data-availability#loc-recommended-repositories.

We wrote public repository (http://hdl.handle.net/...: It is the library of Hokkaido University) relevant DOIs numbers in references, as it possible.

We will update your Data Availability statement on your behalf to reflect the information you provide.

4.Thank you for stating the following in the Financial Disclosure section:

"The authors recieved no specific funding for this work."
We declared no specific funding for this work from Green Consultant Co. Ltd. And Shimadzu Corporation and other companies.

We note that one or more of the authors are employed by a commercial company: Green Consultant Co. Ltd. and Shimadzu Corporation

a) Please provide an amended Funding Statement declaring this commercial affiliation, as well as a statement regarding the Role of Funders in your study. If the funding organization did not play a role in the study design, data collection and analysis, decision to publish, or preparation of the manuscript and only provided financial support in the form of authors' salaries and/or research materials, please review your statements relating to the author contributions, and ensure you have specifically and accurately indicated the role(s) that these authors had in your study. You can update author roles in the Author Contributions section of the online submission form.

Please also include the following statement within your amended Funding Statement.

“The funder provided support in the form of salaries for authors [insert relevant initials], but did not have any additional role in the study design, data collection and analysis, decision to publish, or preparation of the manuscript. The specific roles of these authors are articulated in the ‘author contributions’ section.”

If your commercial affiliation did play a role in your study, please state and explain this role within your updated Funding Statement.

b) Please also provide an updated Competing Interests Statement declaring this commercial affiliation along with any other relevant declarations relating to employment, consultancy, patents, products in development, or marketed products, etc.

Within your Competing Interests Statement, please confirm that this commercial affiliation does not alter your adherence to all PLOS ONE policies on sharing data and materials by including the following statement: "This does not alter our adherence to PLOS ONE policies on sharing data and materials." (as detailed online in our guide for authors http://journals.plos.org/plosone/s/competing-interests) . If this adherence statement is not accurate and there are restrictions on sharing of data and/or materials, please state these. Please note that we cannot proceed with consideration of your article until this information has been declared.

Please include both an updated Funding Statement and Competing Interests Statement in your cover letter. We will change the online submission form on your behalf.

Please know it is PLOS ONE policy for corresponding authors to declare, on behalf of all authors, all potential competing interests for the purposes of transparency. PLOS defines a competing interest as anything that interferes with, or could reasonably be perceived as interfering with, the full and objective presentation, peer review, editorial decision-making, or publication of research or non-research articles submitted to one of the journals. Competing interests can be financial or non-financial, professional, or personal. Competing interests can arise in relationship to an organization or another person. Please follow this link to our website for more details on competing interests: http://journals.plos.org/plosone/s/competing-interests

Additional Editor Comments:

In general, the manuscript contains significant numbers of variables: Therefore, it is advised to present a list of abbreviations for the interest of the readers.

We added The Table of Abbreviation list.

Also, it is advised to shorten the manuscript by elaborating the name of the test method with proper references. Explaining the whole test from samples to test conditions, the brand names of the apparatus again and again has made this important study unnecessary lengthy.

We revised the shorter sentence of test methods as possible as they can.

We avoided to repeat the ^{1}H NMR test and we rewrote the sentence of ^{1}H NMR in text.

Abstract:

Abstract should be concise, with brief problem statement, methodology, results and conclusion. In the current abstract, most of the part is introduction of the problem. It is suggested to exclude large introduction part and concentrate on brief methodology, results and conclusions as per standard format of an abstract.

We added the clear sentence in new introduction.

Introduction:

Line 97: Write the complete name once for “AE”, where it appears for the first time. We revised it.

In general, the introduction part lacks some important literature review related to the study.

I think the Belgian researcher's paper [2] to be the most reliable document for us in the world for concrete damage analysis of delamination and disaggregation. However, here, the cause of the black spots on the back of the concrete floorboard and the white powder on the asphalt pavement surface cannot be explained by the mere snow melting agent, which is ignored even though the SO4- ion is the highest in the chemical analysis. We are greatly appreciated with their paper. The paper helps us to write the paper.

Experiments and test methods:

Line 198: Change the word “Europa” with “Europe”. (I think so).

We revised it.

Line 199: Kindly write full name for “TSM”, “SPNES”, when they appear for the first time OR separately "Provide a list of abbreviations" and

then do not use the whole names throughout the manuscript. At the moment, it is mix-up.

We revised them according to your suggestions.

Line 218: Kindly Write fully explained term for “1H NMR”. This will be otherwise confusing for the readers. You may alternatively write “ Nuclear Magnetic Resonance test”. Similar complete names can be written for 2.2.2, and 2.2.3.

We revised them according to your suggestions.

Line 233: What is meant by “(Themes: 1, 2, 3, 4, 5, 6, 7, 8, 9, 10 ,11) “? Kindly omit all such themes throughout the manuscript as they are not understandable at all.

We diluted those all numbers according to your suggestion.

As a whole, the test procedures are described in detail. In my humble view, brief description with some reference is sufficient for the interest of the readers.

Rather than briefly explaining it with the bibliography Brief, I have the library of Hokkaido university (http://hdl.handle.net/....)in our references. This will allow you to read the paper instantly and will not lengthen the text.

How is it?

Results and Discussion:

It is better to indicate the test methods with reference rather than the brand names of the instrument.

We showed the test method in reference in detail with library of Hokkaido University in our papers.

How is it?

Heading 3.1: Can the observations be presented in tabular form rather than paragraphs?

We revised the “Table” of “results and Discussion”, according to your suggestion.

Line 391: Kindly elaborate “2E1H” for the first time.

We revised it according to your suggestion.

Line 395: What is meant by “This gas”?

It means 2E1H gas. We revised it.

Line 412: Change “investigate” with “investigated”.

Thank you. We revised it. I am ashamed of wrong word.

Kindly elaborate “DD” as “Degree of Deterioration”, when it appears for the first time.

We revised it according to your suggestion.
Kindly enhance font size in Table 1.
Larger tables and fonts will require further table partitioning. For this reason, the size of this font was the limit.
Test methods are repeatedly presented: It is suggested to present the names of the tests and their references rather than explaining the tests again and again. This has made the manuscript very lengthy.
We revised the sentence of ^{1}H NMR test in text.
We revised shorter Test Method.
Conclusions:
Conclusions are somewhat dry. Line 1275 to 1286 can be added to the conclusions rather than being the part of the results and discussion.
We revised all sentence of Conclusions according to your suggestion.

[Note: HTML markup is below. Please do not edit.]

Reviewers' comments:

Reviewer's Responses to Questions

Comments to the Author

1. Is the manuscript technically sound, and do the data support the conclusions?

The manuscript must describe a technically sound piece of scientific research with data that supports the conclusions. Experiments must have been conducted rigorously, with appropriate controls, replication, and sample sizes. The conclusions must be drawn appropriately based on the data presented.

Reviewer #1: Yes
Thank you.

----

2. Has the statistical analysis been performed appropriately and rigorously?

Reviewer #1: I Don't Know
Statistical analysis is not shown in this study.

----

3. Have the authors made all data underlying the findings in their manuscript fully available?

The PLOS Data policy requires authors to make all data underlying the findings described in their manuscript fully available without restriction, with rare exception (please refer to the Data Availability Statement in the manuscript PDF file). The data should be provided as part of the manuscript or its supporting information, or deposited to a public repository. For example, in addition to summary statistics, the data points behind means, medians and variance measures should be available. If there are restrictions on publicly sharing data—e.g. participant privacy or use of data from a third party—those must be specified.

Reviewer #1: Yes
Thank you.

----

4. Is the manuscript presented in an intelligible fashion and written in standard English?

PLOS ONE does not copyedit accepted manuscripts, so the language in submitted articles must be clear, correct, and unambiguous. Any typographical or grammatical errors should be corrected at revision, so please note any specific errors here.

Reviewer #1: No
We have many small errors in sentence. But we revised them.

----

5. **Review Comments to the Author**

Please use the space provided to explain your answers to the questions above. You may also include additional comments for the author, including concerns about dual publication, research ethics, or publication ethics. (Please upload your review as an attachment if it exceeds 20,000 characters)
We agreed with your ethics and publication ethics.

Reviewer #1: Paper is very lengthy and readers won't be able to understand the clear theme of the paper as contents are irregularly arranged.
We have endeavored to keep the text of the paper as short as possible. However, I gave up because if I shortened the text of the paper any longer, I could not explain it in the text.

The authors didn't mention any details regarding 1H NMR, GC-MS (JMS-AX-500), HPLC, High Volume Sampler, One dimensional Transient moisture permeation test and Micro focus CT scanning (CT) in the Introduction section. It's hard for the reader to pick new terms later in the paper.
I believe that this paper would be of interest to highway engineers.
Therefore, make it simple to read.

We added the “Table” of “Abbreviation List”.
----

6. PLOS authors have the option to publish the peer review history of their article (what does this mean?). If published, this will include your full peer review and any attached files.
We agree to your suggestion.

If you choose “no”, your identity will remain anonymous but your review may still be made public.

Do you want your identity to be public for this peer review? For information about this choice, including consent withdrawal, please see our Privacy Policy.
We agreed with your suggestion.

Reviewer #1: No

[NOTE: If reviewer comments were submitted as an attachment file, they will be attached to this email and accessible via the submission site. Please log into your account, locate the manuscript record, and check for the action link "View Attachments". If this link does not appear, there are no attachment files.]

While revising your submission, please upload your figure files to the Preflight Analysis and Conversion Engine (PACE) digital diagnostic tool, https://pacev2.apexcovantage.com/. PACE helps ensure that figures meet PLOS requirements. To use PACE, you must first register as a user. Registration is free. Then, login and navigate to the UPLOAD tab, where you will find detailed instructions on how to use the tool. If you encounter any issues or have any questions when using PACE, please email PLOS at figures@plos.org. Please note that Supporting Information files

do not need this step.

In compliance with data protection regulations, you may request that we remove your personal registration details at any time. (Remove my information/details). Please contact the publication office if you have any questions.
