# Supplementary material for: Deterioration of modern concrete structures and asphalt pavements by respiratory action and trace quantities of organic matter
Source: PLoS One. 2021 May 13;16(5):e0249761. doi: 10.1371/journal.pone.0249761 (PMC8118311; doi:10.1371/journal.pone.0249761)
Supplement: Attachment — Submitted filename: PLOS ONE_Response to reviewers_23thMarch.docx [file pone.0249761.s003.docx]

**PONE-D-20-22508R1**

Modern cement concrete and asphalt pavement are damaged by respiratory

action and trace quantities of organic matter (TQOM): Civil structures

are damaged by respiratory action and trace quantities of organic matter

**Journal Requirements:**

PONE-D-20-22508R2

Deterioration of modern concrete structures and asphalt pavements by respiratory action and trace quantities of organic matter

Dr. akihiro moriyoshi

Dear Dr. moriyoshi,

Thank you for submitting your manuscript entitled "Deterioration of modern concrete structures and asphalt pavements by respiratory action and trace quantities of organic matter" to PLOS ONE. Your manuscript files have been checked in-house but before we can proceed we need you to address the following issues:

1. We note that your manuscript is not formatted using one of PLOS ONE’s accepted file types. Please reattach your manuscript as one of the following file types: .doc, .docx, .rtf, or .tex (accompanied by a .pdf).

If your submission was prepared in LaTex, please submit your manuscript file in PDF format and attach your .tex file as “other.”

We amended old manuscript, new manuscript, Response to reviewers, Cover Letter with Word 2019(DOCX) style. We also amend the sentence of Table 1 and small misses in sentence of new manuscript.

Your manuscript has been returned to your account. Please log on to PLOS Editorial Manager at https://www.editorialmanager.com/pone/ to access your manuscript.

Your manuscript can be found in the "Revisions Sent Back to the Author" link under the New Submissions menu. After you have made the changes requested above, please be sure to view and approve the revised PDF after rebuilding the PDF to complete the resubmission process.

Please note that these changes have been requested to comply with submission guidelines and your manuscript will *not* be sent to review until you have fully adhered to our requests. Once your paper has been seen by an Editor we may return it to you for further information or amendments.

We ask that you address this request within 21 days. If you require additional time, please email the journal office. We are happy to grant extensions of up to one month past this due date. If we have not heard from you within 21 days, your manuscript will be withdrawn from Editorial Manager.

Thank you for submitting your work to PLOS ONE.

Kind regards,

Amy Kiss

PLOS ONE

Please review your reference list to ensure that itis complete and

correct. If you have cited papers that have been retracted, please

include the rationale for doing so in the manuscript text, or remove

these references and replace them with relevant current references. Any

changes to the reference list should be mentioned in the rebuttal letter

that accompanies your revised manuscript. If you need to cite a

retracted article, indicate the article’s retracted status in the

References list and also include a citation and full reference for the

retraction notice.

The reasons for withdrawing the old manuscript and the relationship between the new treatise and the old treatise are shown below.

In addition, I checked all the contents, sentences, tables, etc. of the old manuscript again, corrected all the mistakes, and made it yellow. In Table 1, N / A is newly inserted in the blank.

For the new and old manuscripts, we have created the following list so that you can see the differences in the contents along with the enclosed items, figures, and tables. Table 1 was modified in sentence.

PACE：[moriyosi@eng.hokudai.ac.jp](mailto:moriyosi@eng.hokudai.ac.jp)

Pass word: Moriyoshi828

The new manuscript describes the causes of 11 types of fracture phenomena in asphalt pavement and concrete structures, which were previously unknown, and the old manuscript only describes a few types of fracture phenomena. However, the reason why a part of the contents of the old manuscript (PLSONE-PONE-D-18-30131) was incorporated into the new treatise was that the old manuscript was peer-reviewed by Dr. Andrew Preston, and he said that this treatise was beyond Moriyoshi's imagination. This is because this treatise was said to have great impact. However, he told me that the structure of the treatise and the English text should be viewed by Native person and submitted by the designated date and time. However, when we re-examined the contents of the old treatise, We found that the old treatise had many deficiencies and the structure of the treatise was poor, so it was necessary to make major corrections and it was not possible to meet the deadline. For this reason, we requested the withdrawal of the old treatise at 6:25 AM on 29Nov 2018. I'm very sorry to say that this withdrawal email has already been deleted and there are currently no emails left.

In the new manuscript (PONE-D-20-22508R1), the composition of the treatise, how to organize the treatise, and the English expression were proofread twice by the Native person, so the text was significantly revised. The new treatise also systematically describes the causes of various damages to asphalt pavement and concrete structures over 11 items. The old treatise was only a fragmentary description. For this reason, the new treatise is considered to be a different treatise from the old one. The yellow parts in the table and figures of the new treatise enclosed here are newly added to the new treatise. You can also see from the comparison table of the composition of the treatises that the new and old treatises are completely different treatises.

Unlike the old manuscript, the new manuscript made new discoveries for various deterioration phenomena as follows after the withdrawal of the treatise.

In the new paper, we newly discovered the mechanism of disaggregation, blistering and surface peeling in asphalt pavement, disaggregation in concrete structures, aggregate pop-out and horizontal cracking. It also describes the mechanism by which concrete structures become disaggregation and become fine particles like flour. In addition, asphalt pavement and concrete structures around the world become disaggregation in a short period of time due to trace amounts of organic matter in the air and the breathing action of the structure. The contribution rate of each organic matter for deterioration was also discovered in the new paper.

This indicates the reason why the concrete change like flour due to the collapse of the World Trade Center Building. The new treatise also found that cracks and deterioration are completely different in concrete damage, and that damage depends on the width of the crack and the length of the crack.

In this way, the content and structure of the old treatise has been significantly re-written, so the new treatise will probably be easier for the general reader to understand and be interested in. However, many of the references in the new treatise used similar references to the old ones.

I am very grateful to Dr. Preston for his encouragement and suggestions for the various new discoveries and the structure of the new treatise. We would also like to thank Dr. Khitab and Dr. Magyar for their long-standing advice, proofreading, and support for this treatise.

I think this treatise has become very long and difficult to peer review. We would like to thank all the people concerned.

**Additional Editor Comments (if provided):**

Kindly change the title. Presently, it is in sentence format. Change it

to the title format. May I suggest the following title.

"Deterioration of modern concrete and asphalt pavements by respiratory

action and trace quantities of organic matter". I also think there is no

need for a short title, so that can be omitted.

[Note: HTML markup is below. Please do not edit.]

[NOTE: If reviewer comments were submitted as an attachment file, they

will be attached to this email and accessible via the submission site.

Please log into your account, locate the manuscript record, and check

for the action link "View Attachments". If this link does not appear,

there are no attachment files.]

While revising your submission, please upload your figure files to the

Preflight Analysis and Conversion Engine (PACE) digital diagnostic tool,

https://pacev2.apexcovantage.com/. PACE helps ensure that figures meet

PLOS requirements. To use PACE, you must first register as a user.

Registration is free. Then, login and navigate to the UPLOAD tab, where

you will find detailed instructions on how to use the tool. If you

encounter any issues or have any questions when using PACE, please email

PLOS at figures@plos.org. Please note that Supporting Information files

do not need this step.

In compliance with data protection regulations, you may request that we

remove your personal registration details at any time. (Remove my

information/details). Please contact the publication office if you have

any questions.

**Response to reviewers**

Thank you for your opinion that the title should be a title that seems to be the title, not a sentence. I considered it and made it a title like the following one.

I want concrete to be a structure name, not a substance name.

New title: **Deterioration of modern concrete structures and asphalt pavements by respiratory action and trace quantities of organic matter**
